# Delayed conventional DMARDs therapy is effective in Rheumatoid Arthritis

**DOI:** 10.12669/pjms.334.12704

**Published:** 2017

**Authors:** Tasnim Ahsan, Uzma Erum, Danish Khowaja, Assadullah Dahani

**Affiliations:** 1 Prof. Tasnim Ahsan, MRCP, FRCP, FRCP, FRCP. Jinnah Postgraduate Medical Centre, Medical Unit-II, Rafiqee Shaheed Road, Karachi, Pakistan; 2 Dr. Uzma Erum, MBBS, FCPS Trainee. Jinnah Postgraduate Medical Centre, Medical Unit-II, Rafiqee Shaheed Road, Karachi, Pakistan; 3 Dr. Danish Khowaja, MBBS, FCPS Trainee. Jinnah Postgraduate Medical Centre, Medical Unit-II, Rafiqee Shaheed Road, Karachi, Pakistan; 4 Dr. Assadullah Dahani, MBBS, FCPS. Jinnah Postgraduate Medical Centre, Medical Unit-II, Rafiqee Shaheed Road, Karachi, Pakistan

**Keywords:** CDAI, Disease activity, DMARDs, Rheumatoid arthritis

## Abstract

**Objective:**

To determine the disease severity in patients with Rheumatoid Arthritis (RA), at baseline and the impact of treatment on disease activity (DA) after six months of disease modifying anti-rheumatic drugs (DMARDs) therapy.

**Methods:**

This prospective study was conducted at the ‘Rheumatology Clinic’ of Jinnah Postgraduate Medical Centre (JPMC), Karachi, from June 2014 to May 2015. A total of 111 patients, with the diagnosis of RA were included in the study. DA was calculated using ‘Clinical Disease Activity Index’ (CDAI) score at base line and after 6 months of DMARDs therapy.

**Results:**

Out of 111 patients, 17 (15.3%) were male and 94 (84.7%) were female. The mean age was 37.16±11.3 years and the mean duration of joint pain was 3.8±3.6 years (median 2.5 years). The mean Hb was 10.8±1.8 g/dl and the mean ESR at baseline was 59.63±30.9 mm/Hr. The mean initial CDAI score was 18.14±11.69; reflecting moderate to severe disease. Of all of these patients, 32 (28.8%) patients received monotherapy, 78 (70.3%) received dual therapy and 1(0.9%) was given triple DMARDs therapy. The mean ESR was 39.5±27.31 mm/Hr, and mean CDAI was 7.36±7.8 with a median of 6.0 after 6 months of DMARDs treatment.

**Conclusion:**

The CDAI score and the ESR reflected that majority of our patients were in remission or at low disease activity, after six months of DMARDs therapy. It is possible to control DA in RA, in a low resource health care facility with conventional DMARDs therapy. Continuity of treatment was ensured through motivation, regular supply of drugs and regular follow-up.

## INTRODUCTION

Rheumatoid arthritis (RA) is a multisystem inflammatory disorder, which has a significant negative impact on an individual’s quality of life and activities of daily living. Current recommendations focus on an early aggressive therapy in accordance with disease activity (DA) to achieve remission.^1^ The use of composite disease activity tools in RA help in prompt and early treatment, in order to halt disease progression. Several indices for the assessment of disease activity (DA) have been defined by American College of Rheumatology (ACR); these include Routine Assessment of Patient Index Data 3 (RAPID3), Clinical Disease Activity Index’ (CDAI), Simplified Disease Activity Index (SDAI) and Disease Activity Score 28 (DAS28).^2^ CDAI was used as a tool for routine assessment of DA in patients in our clinic. There has been a rapid addition of biological drugs for the treatment of RA over last decade. However, treatment with biological agents is quite expensive and entails potential long term side effects. Hence, Methotrexate (MTX) is considered to be the first line DMARD to initiate RA treatment.^3^ The aim of this study was to ascertain the outcome of treatment in terms of improvement in DA and to halt the progression of disease via the use of conventional DMARDs in patients with RA.

## METHODS

### Study Design

This was a prospective, longitudinal study conducted at the ‘Rheumatology Clinic’ of Jinnah Postgraduate Medical Centre (JPMC), from June 2014 to May 2015.

### Inclusion Criteria

A total of 111 patients with the diagnosis of RA, according to the ACR criteria, of any age and sex were included in the study, who were either treatment naïve or had previously taken erratic and sub-optimal DMARDs therapy.

### Exclusion Criteria

Patients with poor drug compliance and irregular follow-up visits to the clinic were not included in the study, as were those already taking DMARDs treatment.

### Data Collection and Analysis

Detailed history and examination was recorded, DA was calculated using CDAI. Laboratory investigations, including CBC, RA factor, ESR, ALT and creatinine were done. All the data as well as the treatment regimen were recorded in a pre-designed structured proforma. All the patients were regularly supplied with the prescribed drugs. Data was analyzed by SPSS version-17. For the descriptive variables like gender, DA and treatment regimen, frequency and percentages were calculated, while means were calculated for age, duration of disease and CDAI score.

## RESULTS

Out of the 111 patients 17 (15.3%) were male and 94 (84.7%) were female. The mean age was 37.16±11.3 years and the mean duration of joint pain was 3.8±3.6 years (median 2.5 years). Most patients were in the age group of 21-40 years i.e. 71 (63.9%) patients. The mean Hb was 10.8±1.8 g/dl and the mean ESR at baseline was 59.63±30.9 mm/Hr. Out of the 111 patients 43 (38.7%) were treatment naïve, 48 (43.24%) were regular NSAIDs user, 3 (2.7%) were on hakeemi medications and 17 (15.31%) patients were on erratic DMARDs.

**Fig. 1:**
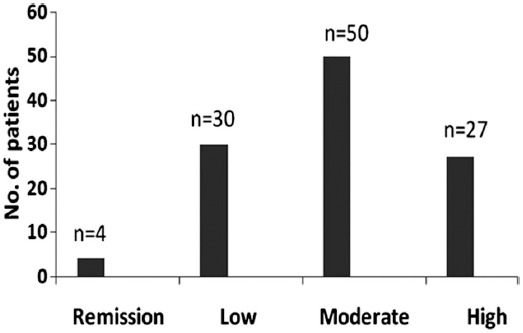
Disease activity at baseline.

**Fig. 2:**
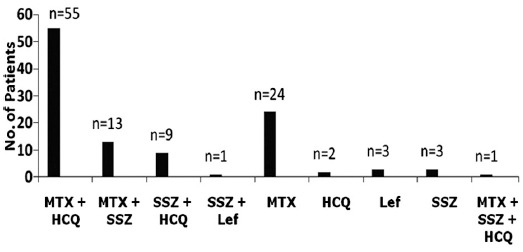
Treatment regimen in RA patients.

**Fig. 3:**
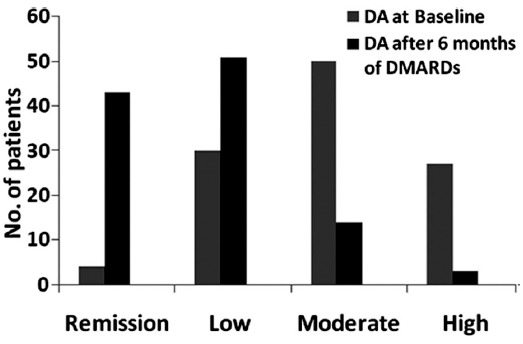
Disease activity, before and after 6 months of DMARDs therapy.

Disease activity was calculated by using CDAI, and patients and patients were grouped into remission 4 (3.6%) patients, low DA 30 (27.0%) patients, moderate DA 50 (45.0%) patients and severe DA 27 (24.3%) patients. (Fig. 1). The mean initial CDAI score was 18.14±11.69 that reflected moderate to severe disease at the time of presentation in our clinic. With regard to treatment, DA of individual patient was used as a parameter for treatment regimen i.e patients with moderate or high DA were given either dual or triple DMARDs therapy, while those with low DA or those in remission were given monotheray. Among all, 32 (28.8%) patients received monotherapy, 78 (70.3%) received dual therapy and 1(0.9%) was given triple DMARDs therapy, i.e MTX, Hydroxycholorquin (HCQ) and Sulphasalazine (SSZ) (Fig. 2). Usual treatment regimen used was MTX and Hydroxychloroquine (HCQ) in 55 (49.5%) patients(Fig. 2). MTX was used in a dose of 10-12.5 mg/week in most patients; however, few patients required a dose of up to 20 mg/week. Short term low-dose systemic steroids were used in 41 (36.9) patients, intra-articular steroids in one or two joints in 14 (12.6%) patients, both systemic and intra-articular steroids were given in 8 (7.2%) patients. All the patients were regularly followed-up in the clinic at 4 weekly intervals. After six months of regular treatment CDAI and ESR were re-evaluated. The mean ESR was 39.5±27.31 mm/Hr, and mean CDAI was 7.36±7.8 with a median of 6.0, after 6 months of DMARDs treatment. With regard to DA, 43 (38.7%) patients achieved remission, 51 (45.9%) were in low DA, 14 (12.6%) were in moderate DA and 3 (2.7%) patients remained in high DA, after receiving 6 months of DMARDs treatment. Thus, overall 94 (84.6%) patients either achieved remission or low DA after regular DMARDs therapy. A comparison of DA at baseline and after 6 months of DMARDs therapy is shown in Fig. 3.

## DISCUSSION

The present study demonstrates the outcome of using DMARDS in patients with rheumatoid arthritis. A major stride in treatment regimen of RA has been seen over the past decades with the advent of biological agents. These advancements in the therapeutic strategies of RA have led to an early and more effective disease control. However, for poor countries like Pakistan, the cost of biological agents is prohibitive. Serial objective assessment of DA in RA is imperative to evaluate disease remission. Remission can be achieved in most patients with the use of conventional DMARDs, provided an appropriate compliance can be maintained by motivation, through provision of drugs and regular follow-up.

As higher DA in RA is associated with greater joint damage and poor outcome in terms of joint integrity and function, DMARDs therapy aims at achieving quick remission.^4^ Increased joint damage over time has been reported, even with low disease activity.^5^,^6^ Nonetheless, remission as a reflection of either ‘no or minimal disease activity’ ought to ensure prompt disease control and preservation of joint function and quality of life. CDAI appears to be a stringent measure that allows for only minimal residual joint count for swelling and tenderness, as a marker of disease remission. Hence it is not inappropriate to use CDAI as an assessment tool for DA in clinical setting. A study from India reported similar efficacy of DAS28 and CDAI to RAPID3, even though RAPID3 does not include formal joint count.^7^ It has already been reported that CDAI and DAS28 have a good co-relation for DA assessment in RA patients.^8^ In addition, CDAI can be easily used in a clinical setting without the help of calculator and laboratory values for inflammatory markers.

In this study, all patients were treated with traditional DMARDs, mainly MTX, thus reflecting a considerable potential to achieve remission in RA patients even with conventional treatment regimen, used timely and in appropriate doses. The efficacy of conventional DMARDs alone has been reported in other studies as well.^9^ In addition to MTX, patients were treated with different drug combinations in our study. It has been suggested that MTX is a valuable treatment option in patients who require combination DMARDs treatment or failed on monotheray.^10^ No significant difference has been reported previously, in the outcome of treatment with MTX alone or other DMARDs.^11^ However, intensive MTX doses have been reported to substantially enhance the efficacy of treatment and sustained remission.^12^ Majority of our patients required a dose of 10-12.5 mg/wk of MTX, and adjustments in MTX dosage were tailored to individual patients. However, not all patients responded equally to this treatment, because of varying DA and different stages of progression of RA. The possible factors such as disease duration and its severity, impact of environmental factors and genetic make-up, all contribute towards an individual’s response to therapy. It has been reported that subcutaneous use of MTX provides excellent tolerability and compliance with equivalent efficacy to oral treatment.^13^

In addition, the rate of sustained remission needs to be evaluated, as varying DA might cause some patients to fulfill criteria for remission at different points in treatment course. It has been suggested that the therapeutic response in the first 3 months of therapy predicts the potential to achieve remission.^14^ This study cohort shows a considerable number of patients achieving remission over a period of 6 months. Hence it can be postulated that these patients would likely maintain a remission in due course of time, as long treatment integrity and compliance is maintained.

## CONCLUSION

This study represents a small cohort of patients who attained disease remission with the use of conventional drugs. Hence, it supports the notion that a cost effective treatment strategy with the use of traditional DMARDs can achieve control of DA and hence preserve joint integrity, provided appropriate compliance can be ensured.

### Author’s Contribution

**TA:** Conception and design, drafting and revision of the article, final approval of the manuscript.

**UE:** Conception and design, acquisition, analysis and interpretation of data, drafting and revision of the article, final approval of the manuscript.

**DK:** Conception & design, acquisition & analysis of data, drafting the article, final approval of the manuscript.

**AD:** Conception and design, drafting and revision of the article, final approval of the manuscript.
